# Correlation between metastatic patterns and age in patients with metastatic primary liver cancer: A population-based study

**DOI:** 10.1371/journal.pone.0267809

**Published:** 2023-01-27

**Authors:** Zhaoting Zheng, Yue Hu, Yutong Ren, Guoheng Mo, Hao Wan

**Affiliations:** 1 Department of Neurosurgery, Beijing TianTan Hospital, Capital Medical University, Beijing, China; 2 Queen Mary College of Nanchang University, Nanchang, Jiangxi, China; 3 Department of Infectious Diseases, Southern Hospital, Southern Medical University, Guangzhou, China; 4 Hepatobiliary Surgery Department, The First Affiliated Hospital of Nanchang University, Nanchang, Jiangxi, People’s Republic of China; Texas Tech University Health Science, Lubbock, UNITED STATES

## Abstract

**Aim:**

Primary liver cancer is usually diagnosed at advanced stages with distant metastasis, underlying the high metastatic rate and mortality in patients. This study aimed to analyse the metastatic patterns and prognosis of primary liver cancer, and its relationship with age and several other factors, such as histological variants, TNM stage, and grade.

**Methods:**

We included data from 5274 patients from the Surveillance, Epidemiology, and End Results (SEER) database of the American National Cancer Institute diagnosed with primary liver cancer with metastatic disease between 2010 and 2015. The correlation between the metastatic patterns of primary liver cancer and age was evaluated. The hazard ratio (HR) and 95% confidence intervals (CI) for overall survival were calculated by applying univariate Cox analysis, while the correlation between the metastatic patterns and age was analysed by applying multivariate Cox analysis. We also plotted Kaplan-Meier curves to illustrate the correlation between overall survival (OS) and various factors.

**Results:**

Several factors were associated with poorer prognosis, including age>60 years, histologic type of spindle cell variant, higher grade, no surgery, tumour size ≥ 1 cm, and lung metastasis. The rate of metastasis increased with age. Older patients (> 50 years) were prone to bone metastasis, while less likely to have lung metastasis compared with younger patients (< 50 years). Patients with lung metastasis had a higher risk of being diagnosed with metastasis in other locations. Furthermore, surgery significantly reduced mortality and primary site surgery in particular, mitigated the risk of bone and lung metastases.

**Conclusions:**

Our study shows the correlation of prognosis and metastatic patterns with age and several other factors. The findings can hopefully provide knowledge that will allow a better diagnosis and management of elderly patients with primary liver cancer.

## Introduction

Liver cancer is the fourth most common cause of cancer-related deaths (CRD) worldwide [1]. It exhibits the second highest mortality rate globally, with a 5-year survival rate of 18% [2, 3]. Hepatocellular carcinoma (HCC) and intrahepatic cholangiocarcinoma (iCCA) are the two major types of primary liver cancers, accounting for over 90% of all cases [4]. Primary liver cancers are usually diagnosed in patients with chronic liver diseases, including hepatocellular damage attributed to fatty liver disease and viral infections. However, the application of HBV vaccination and hepatitis C direct antiviral drugs have significantly reduced the number of virus-induced liver cancers; hence, non-alcoholic fatty liver disease (NAFLD) is becoming the main cause of liver cancer [5].

In the last decade, the mortality rate of liver cancer among men and women in the United States has increased, especially among those aged over 50 years [6]. The treatment of most liver cancers consists of trans-arterial chemoembolization, oral administration of sorafenib, or immunotherapy. However, long-term use of chemotherapy, such as sorafenib, commonly exerts toxic effects [7], while surgical treatment is an option only for 5% to 15% of patients, diagnosed at an early stage [8]. Moreover, elderly patients are prone to postoperative complications [9]. Thus, given the delicate balance between risks and benefits, the selection of therapeutic regimens that benefit elderly liver cancer patients represents a dilemma.

Aging has been associated with a poorer prognosis in liver cancer [10, 11]. A significantly higher mortality rate has been observed in the foreign-born population compared with the US-born population [6]. Different histologic variants, surgical treatments, and location of metastasis also result in different prognoses and metastatic patterns of liver cancer [12, 13]. Such differences could be due to changes in lifestyle, presence of HBV infection, or the metabolic changes of aging. Furthermore, the poorer prognosis related to age and to some histologic variants, such as the spindle cell variant, could be related to the rate of metastasis. Thus, we believe it is worth evaluating the metastatic patterns, survival outcomes, and their association with age.

Existing studies have mostly focused on the pathogenesis and mechanisms of liver cancer. A study by Wu et al. demonstrated that different liver cancer histological variants had different metastatic patterns [14]. However, to the best of our knowledge, the correlation between liver cancer metastasis, mortality, and aging has not been investigated. Based on the data of adult primary liver cancer patients in the Surveillance, Epidemiology, and End and Result (SEER) database, this study was performed on factors including age, sex, race, grade, T stage, N stage, and histological type, etc. There are two main aims: 1) determine which parameters influence metastatic pattern of either bone, brain or lung; 2) determine which parameters influence overall survival (OS) The present study attempted to provide insights into the prognosis of primary liver cancer among patients with different conditions, thereby seeking to inspire future treatment groups.

## Materials and methods

### Ethics

All data of cancer patients were acquired from the SEER database (https://seer.cancer.gov/data/access.html) with the reference number: 13003-Nov2019. SEER is a public database, and no ethical approval is needed. Informed consent was not required. The data of individual participants was accessible from the SEER database.

### Data collection

SEER* Stat version 8.3.4 was used to filter the data of the patients from the SEER database (http://seer.cancer.gov/). Patient selection complied with the SEER 18 Regs Research Data, as maintained by the National Cancer Institute. The inclusion criteria of this study were as follows: 1) patient age ≥ 18 years old; 2) primary site: liver; 3) availability of information regarding histological subtypes (histologic type ICD-O-3: 8170–8174); 4) malignant behaviour; 5) primary liver cancer confirmed by biopsy or microscopy; and 6) diagnosis performed between 2010 and 2015. Patients with unknown survival status and survival times were excluded, as well as those not at the M1 stage, according to the TMN staging system. Individual information can be assessed in the SEER database. As listed in Table 1, the clinical characteristics analyzed in the current study included age, race, sex, year of diagnosis, histologic type, grade, laterality, T stage, N stage, primary site surgery, other site surgery, lymph node surgery, bone metastasis, lung metastasis, liver metastasis. Laterality was defined as location of primary tumor. OS was defined as the period between diagnosis and death by any cause as described in SEER database.

**Table 1:** Characteristics of patients.

Clinicopathological Characteristics	All patients n = (5,274)	Bone n = (1551)	Brain n = (116)	Lung n = (2140)
	No (%)	No (%)	No(%)	No(%)
**Age**				
Age<50	374(7)	71(5)	11(10)	191(9)
Age50-59	1615(31)	472(30)	38(32)	650(30)
age≥60	3285(62)	1008(65)	67(58)	1299(61)
**Sex**				
Male	4281(81)	1326 (85)	94(81)	1687(79)
Female	993(19)	225(15)	22(19)	453(21)
**Race**				
Black	842(16)	276(18)	19(16)	372(17)
White	3591(68)	1079(69)	82(71)	1356(63)
Other	824(15)	191(12)	14(12)	403(19)
Unknown	17(1)	5(1)	1(1)	9(1)
**Year diagnosis**				
2010	802(15)	203(13)	18(16)	303(14)
2011	810(15)	232(15)	21(18)	326(15)
2012	900(17)	284(18)	14(12)	362(17)
2013	912(17)	277(18)	24(21)	373(17)
2014	897(17)	253(16)	17(14)	382(18)
2015	953(19)	302(20)	22(19)	394(18)
**Histologic type**				
8170(NOS)	5188(98)	1532(99)	115(99)	2100(98)
8171(fibrolamellar)	27(<1)	3(<1)	0	13(1)
8172(scirrhous)	6(<1)	1(<1)	0	2(<1)
8173(spindle cell variant)	18(<1)	4(<1)	0	7(<1)
8174(clear cell variant)	35(1)	11(1)	1(1)	18(8)
**Grade**				
I	312(6)	100(6)	11(10)	104(5)
II	595(11)	149(10)	21(18)	246(12)
III	610(12)	143(9)	9(8)	278(13)
IV	61(1)	9(1)	3(3)	22(1)
Unknown	3696(70.0)	1150(74)	72(62)	1490(70)
**Laterality**				
Paired	10(<1)	0	0	5(<1)
Not paired	5264(100)	1551(100.0)	116(100.0)	2135(100)
**T stage**				
T0	28(<1)	16(1)	2(2)	11(1)
T1	1071(20)	330(21)	34(29)	433(20)
T2	547(10)	184(12)	14(12)	196(9)
T3a	1058(20)	328(21)	19(16)	425(20)
T3b	870(17)	194(13)	10(9)	327(15)
T3NOS	11(<1)	2(<1)	0(0)	5(<1)
T4	544(10)	97(6)	7(6)	256(12)
TX	1145(22)	400(26)	30(26)	487(23)
**N stage**				
N0	3132(59)	984(63)	78(67)	1321(62)
N1	1236(24)	286(19)	15(13)	425(20)
NX	906(17)	281(18)	23(20)	394(18)
**Primary site surgery**				
Yes	178(3)	39(3)	5(4)	45(2)
No	5096(97)	1512(97)	111(96)	2095(98)
**Lymph node surgery**				
Yes	46(1)	7(1)	0(0)	10(1)
No	5228(99)	1544(99)	116(100)	2130(99)
**Other surgery**				
Yes	209(4)	110(7)	12(10)	52(3)
No	5065(96)	1441(93)	104(90)	2088(97)
**Tumor size**				
<1cm	3730(71)	1069(69)	78(67)	1472(69)
≥1cm	4(<1)	1(<1)	0(0)	2(<1)
Unknown	1540(29)	481(31)	38(33)	666(31)
**Bone**				
Yes	1551(29)		44(38)	353(17)
No	3491(66)		69(60)	1721(80)
Unknown	232(5)		3(2)	66(3)
**Brain**				
Yes	116(2)	44(3)		47(2)
No	4871(92)	1446(93)		2015(94)
Unknown	287(6)	61(4)		78(4)
**Lung**				
Yes	2140(41)	353(23)	47(41)	
No	2886(55)	1135(73)	66(57)	
Unknown	248(4)	63(4)	3(3)	

Note: NOS, non-specific hepatocellular carcinomas.

### Statistical analysis

The effect of each clinical feature on overall survival (OS) was identified using hazard ratio (HR) and 95% confidence intervals (CIs). For nomogram model construction, univariate Cox regression was performed to screen the factors that affected the overall survival, and multivariate Cox regression was performed to further screen the independent prognostic factors. Then, the factors with P < 0.05 in the multivariate Cox regression analysis were included in the nomogram model. The predictive performance of nomogram model was assessed by the consistency index (C-index). C-index of 0.5 represent a random probability, indicating that this model has no predictive effect, while C-index of 1 represent a complete consistent, indicating that the predicted results of the model are completely consistent with the reality [15]. Age as a clinical feature was categorized into three groups (≤ 50 years, 51–59 years, and >60 years). This grouping pattern follows the same general grouping pattern used in other cancer studies [14]. Additionally, these groupings lead to the outcome which were identified as more statistically significant. Logistic regression models were adopted to assess the correlation between the metastatic patterns and clinical features. The relationship between the risk of metastasis with a variety of factors was analysed using the odds ratio (OR). The curve of the metastatic pattern was generated using SPSS 21.0. The Kaplan-Meier analysis method was employed for survival curves, followed by chi-square test for statistical significance. Statistical significance was defined as P < 0.05 for all tests. The multivariate Cox regression and all calculations were carried out using R version 3.2.3. The R packages used in this study including RMS, foreign, and survival package.

## Results

### Demographic characteristics

After applying the aforementioned inclusion criteria, a total of 5274 liver cancer patients were included in the study (Table 1), of which 4281 were men (81.1%) and 993 women (18.8%). The majority of the patients were aged over 60 years (<50 years, 374, [7%]; 50–59 years, 1615, [30.6%]; ≥60 years, 3285, [62.1%]) and White (White, 3591, [68%]; Black, 842, [15.9%]; Other, 824, [15.6%]; Unknown, 17, [0.3%]). Regarding histological variants, 98.3% of patients were diagnosed with non-specific hepatocellular carcinomas (NOS). For tumor size, there were only 4 samples (< 1%) with tumor size ≥ 1cm among the 5274 samples. The majority of samples had tumor size < 1cm. Additionally, most of the patients did not undergo surgical treatment (91.8%). The distant metastasis sites were primarily the lungs (2140, 40.6%), followed by the bones (1551, 29.4%); brain metastases were rare (n = 116, 2.2%).

### Metastasis patterns

**Fig 1:**
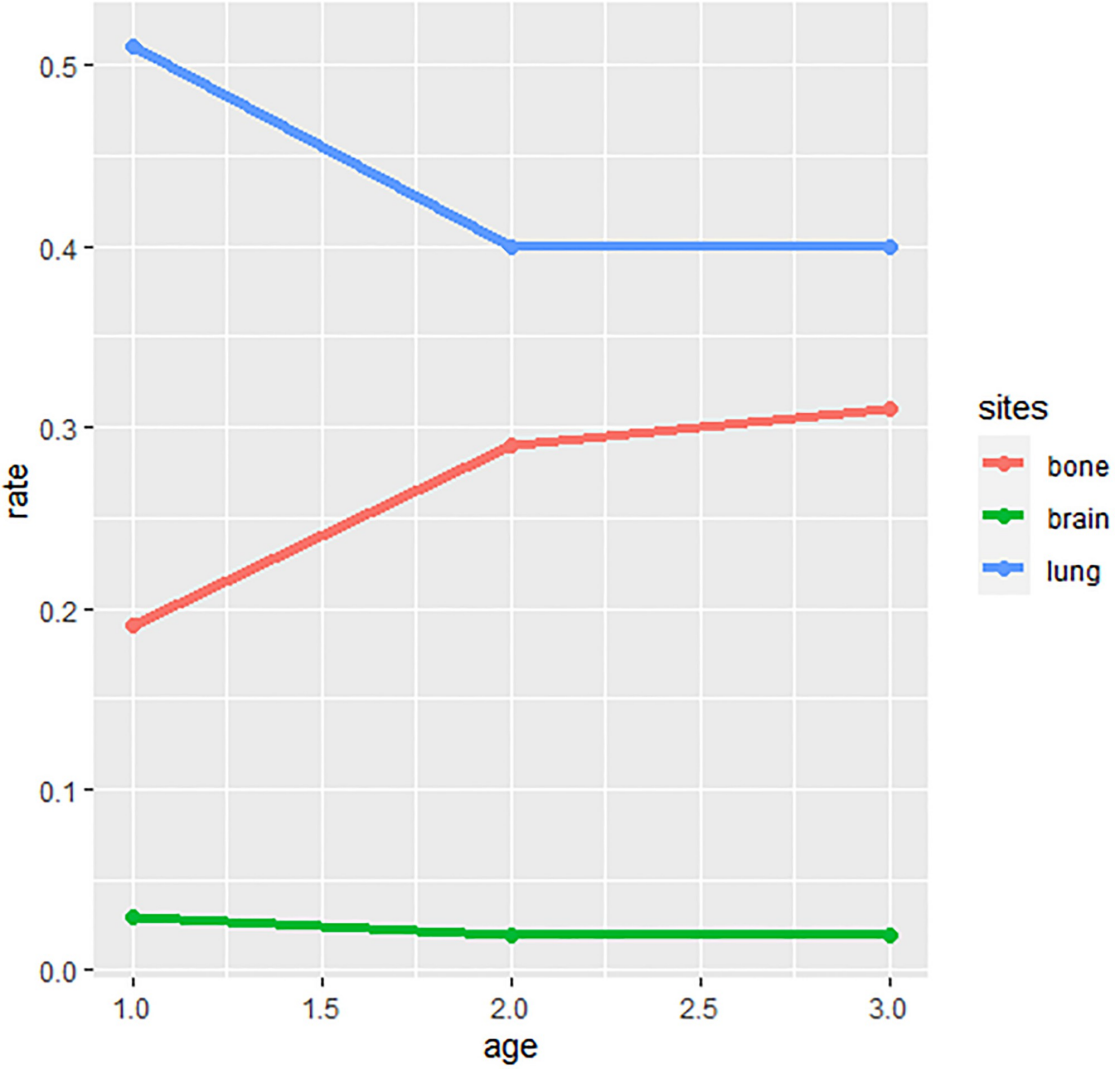
Rate of distant metastatic sites associated with age. The line chart shows the metastasis rate of bone, brain and lung at three age groups (< 50 years, 50–59 years and ≥ 60 years). Y-axis represent metastasis rate and X-axis represent age groups.

The analysis of different metastasis sites showed that the risk of developing metastatic disease in bones increased with age, whereas it is opposite in brain and lungs (Fig 1). Age ≥ 60 years was found to be associated with the maximum risk of metastasis at bones, and age at 50–59 and ≥ 60 had lower risk of metastasis at lung. Whereas brain metastasis was found to be the lowest and similar among the 3 age groups. Table 2 showed the proportion of metastases patterns in different age group. It could been seen that the proportion of patients with bone metastases showed an increasing trend with age (0.190, 0.290 and 0.310 in different age groups). There was no difference in the proportion of patients with brain metastasis among different age groups. Patients (< 50 years) had a relatively high proportion of lung metastases (0.510 vs. 0.40).

**Table 2:** The proportion of metastases patterns in different age group.

Age	Bone metastasis		Brain metastasis		Lung metastasis	
	Yes	Proportoin	Yes	Proportoin	Yes	Proportoin
<50	71	0.19	11	0.03	191	0.51
50–59	472	0.29	38	0.02	650	0.4
≥60	1008	0.31	67	0.02	1299	0.4

According to the results of logistic regression, various factors were relevant to the metastatic patterns (Table 3). Patients older than 50 years are more likely to develop bone metastases (for 50–59 year: OR = 1.505, p = 0.007; for ≥ 60 years: OR = 1.669, p<0.001), while had a low risk of lung metastases (for 50–59 year: OR = 0.743, p = 0.017; for ≥ 60 years: OR = 0.716, p = 0.005). Compared with Blacks, Whites and other ethnicities had a low risk to develop bone metastases (for Whites: OR = 0.773, p = 0.003; for Other: OR = 0.629, p<0.001), and whites also had a low risk to develop lung metastases (OR = 0.730, p<0.001). Males are more likely to develop bone metastases (OR = 1.484, p<0.001) and had a low risk to develop lung metastases (OR = 0.846, p = 0.026) than females. Patients with advanced grade had increased risk to develop lung metastasis (OR for Grade II, III and V were 1.378, 1.656, 1.341, respectively; P<0.050), however, there was no statistical significance for Grade IV (P = 0.970). In terms of T stage, T1-T4 showed a relatively low risk of bone metastasis than T0 (OR<1, P<0.050). Compared with N0, N1 had a low risk of bone metastasis (OR = 0.649, P<0.001) and lung metastasis (OR = 0.651, P<0.001). The risk of bone metastases (OR = 1.868, P = 0.002) and lung metastases (OR = 2.326, P<0.001) is relatively high in those who have not undergone primary site surgery, while the risk of bone metastases (OR = 0.362, P<0.001) and brain metastases (OR = 0.374, P = 0.003) is decreased in those who have not undergone other site surgery. In addition, the patients without brain metastasis had an high risk to develop bone metastasis (OR = 3.387, P<0.001), while the patients without lung metastasis had low risk to develop bone metastasis (OR = 0.698, P = 0.010).

**Table 3:** Logistic regression of metastasis patterns.

Clinicopathological Characteristics	Bone metastasis		Brain metastasis		Lung metastasis	
	OR	Pr(>|z|)	OR	Pr(>|z|)	OR	Pr(>|z|)
**Age**						
<50	reference	reference	reference	reference	reference	reference
50–59	**1.5047**	**0.0074**	0.7218	0.3578	**0.7429**	**0.0169**
≥60	**1.6692**	**0.0005**	0.5717	0.0968	**0.7164**	**0.0048**
**Race**						
Black	reference	reference	reference	reference	reference	reference
White	**0.7731**	**0.0033**	1.0189	0.943	**0.7301**	**0.0001**
Other	**0.629**	**0.0001**	0.7907	0.5159	1.0738	0.4947
Unknown	0.8261	0.7416	2.4161	0.4125	1.2515	0.665
**Sex**						
Female	reference	reference	reference	reference	reference	reference
Male	**1.4835**	**<0.0001**	0.9551	0.8503	**0.8455**	**0.0258**
**Histologic types**						
NOS	reference	reference	reference	reference	reference	reference
Fibrolamellar	0.6785	0.5512	0	0.99	1.1842	0.698
Scirrhous	0.998	0.9986	0	0.9954	0.6125	0.5799
Spindle cell variant	0.8412	0.7729	0	0.9923	1.0899	0.8669
Clear cell type	1.5719	0.2459	1.04	0.9698	1.7199	0.1363
**Grade**						
Grade I	reference	reference	reference	reference	reference	reference
Grade II	0.7704	0.1078	1.0383	0.9221	**1.3781**	**0.0364**
Grade III	0.8254	0.2398	0.4319	0.0685	**1.6559**	**0.0009**
Grade IV	**0.3854**	**0.0165**	1.4404	0.5903	0.9888	0.9704
Grade V	1.0619	0.6525	0.5309	0.0579	**1.3414**	**0.0245**
**Laterality**						
Paired	reference	reference	reference	reference	reference	reference
Not Paired	NA	0.9366	NA	0.9941	0.9939	0.9925
**T Stage**						
T0	reference	reference	reference	reference	reference	reference
T1	**0.3697**	**0.0156**	0.4193	0.2576	0.8162	0.6237
T2	**0.4174**	**0.0359**	0.3427	0.1778	0.7306	0.4538
T3a	**0.4025**	**0.0269**	0.2566	0.082	0.8311	0.6549
T3b	**0.2379**	**0.0005**	**0.1732**	**0.032**	0.6722	0.3399
T3NOS	0.1877	0.0645	0	0.9935	0.9609	0.9581
T4	**0.2081**	**0.0002**	0.1972	0.0553	0.9693	0.9407
TX	**0.4528**	**0.0582**	0.3415	0.1807	0.8994	0.8005
**N stage**						
N0	reference	reference	reference	reference	reference	reference
N1	**0.6491**	**<0.0001**	0.606	0.0848	**0.6509**	**<0.0001**
NX	1.031	0.7416	1.1396	0.6143	1.0836	0.3476
**Primary site surgery**						
Yes	reference	reference	reference	reference	reference	reference
No	**1.8678**	**0.0019**	0.9655	0.9427	**2.3264**	**<0.0001**
**Lymphnode surgery**						
Yes	reference	reference	reference	reference	reference	reference
No	2.3642	0.053	NA	0.9871	1.8866	0.0963
**Other site surgery**						
Yes	reference	reference	reference	reference	reference	reference
No	**0.3623**	**<0.0001**	**0.3738**	**0.0028**	**1.4976**	**0.0199**
**Bone metastasis**						
Yes			reference	reference	reference	reference
No			0.9597	0.8431	3.3995	0
Unknown			0.5857	0.4313	1.4101	0.0875
**Brain metastasis**						
Yes	reference	reference			reference	reference
No	**3.387**	**<0.0001**			0.8338	0.2071
Unknown	**1.9211**	**0.0005**			1.4464	0.7267
**Lung metastasis**						
Yes	reference	reference	reference	reference		
No	**0.6977**	**0.01**	0.795	0.2825		
Unknown	0.8043	0.8612	0.6233	0.4911		
**Tumour size**						
<1cm	reference	reference	reference	reference	reference	reference
≥1cm	1.067	0.4899	NA	0.9965	1.121	0.175
Unknown	NA	0.9325	1.0869	0.7572	**0.1193**	**0.0213**

Note: NOS, non-specific hepatocellular carcinomas.

### Survival analysis

**Fig 2:**
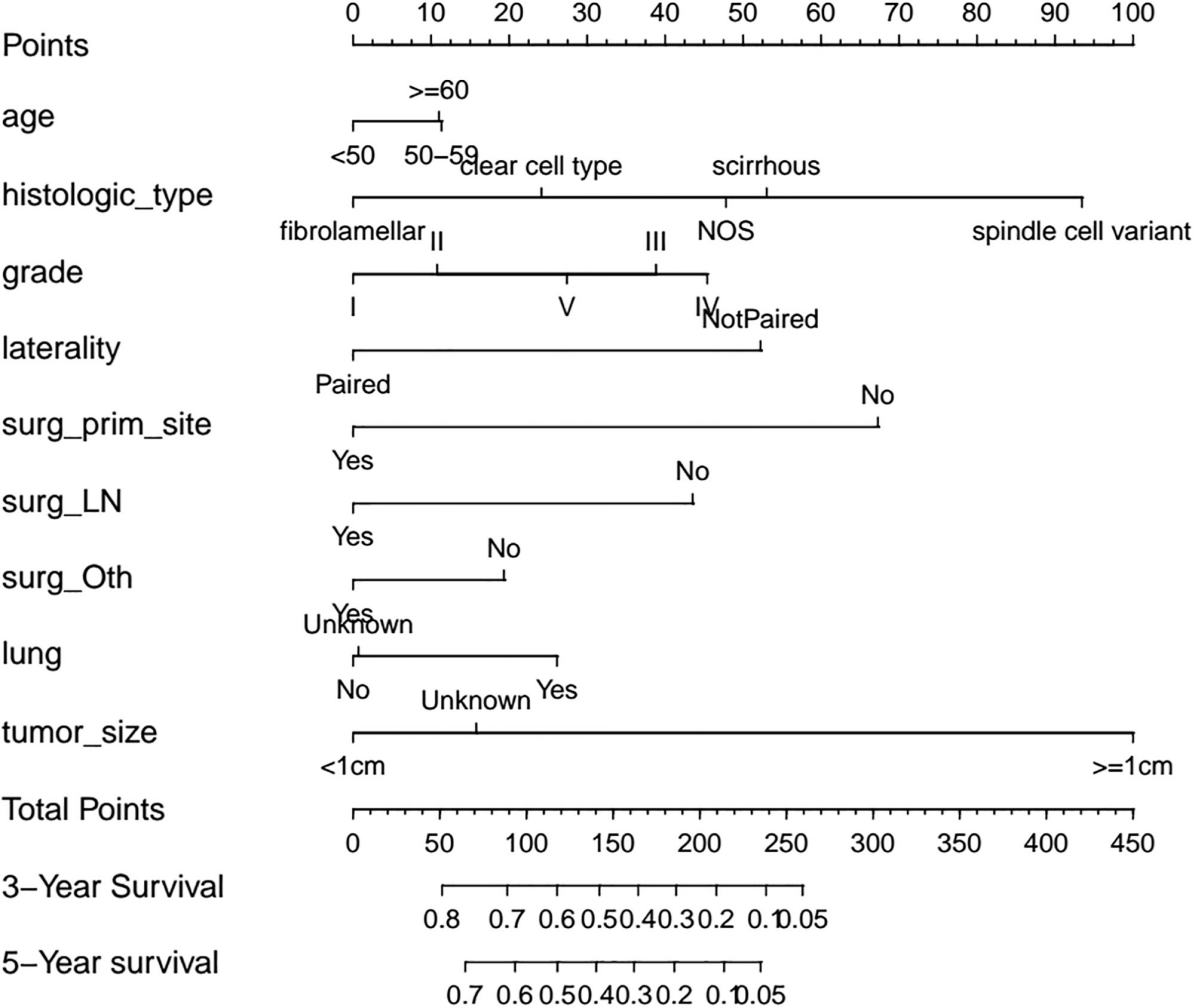
Nomogram model. The Nomogram model was established by 9 independent prognostic factors, which could predict the 3-year and 5-year survival for patients. In which histologic type 8173 (spindle cell variant) corresponds to maximum points for risk of death, followed by no primary site surgery.

**Fig 3:**
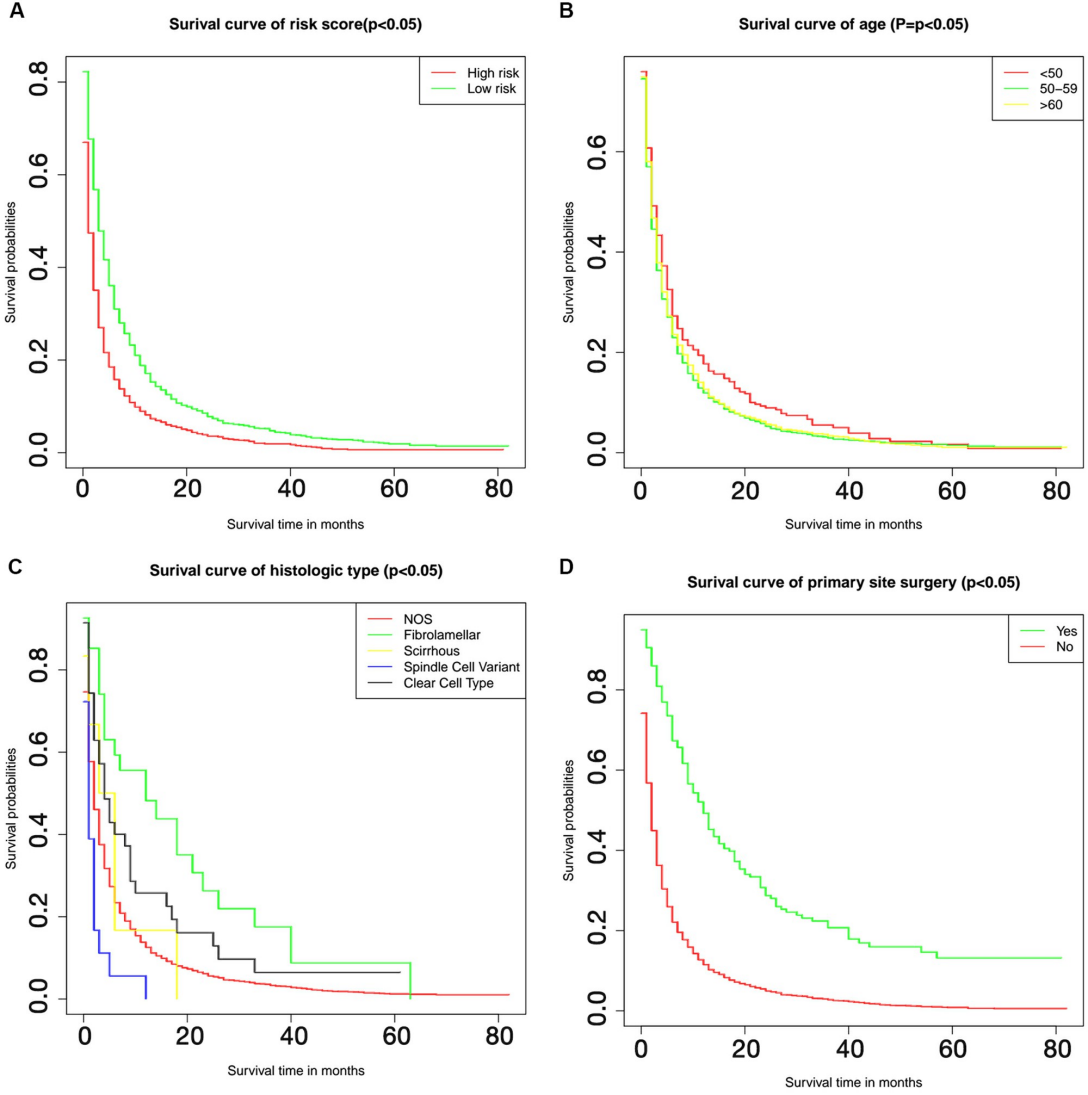
Kaplan-Meier curves based on different factors. The Kaplan-Meier survival curves show the differences on survival probabilities within high and low risk (A), among three age groups (B), among five histologic types (C) and whether received primary site surgery or not (D).

In the univariate Cox regression analysis, a variety of factors were found to be associated with OS of patients, including histologic types, grade, T stage, N stage, primary site surgery, lymph node surgery, other site surgery, bone metastasis, lung metastasis and tumor size (Table 4). For the multivariate Cox regression analysis, a variety of factors were identified as independent risk factors (p<0.05) correlated to OS, including age, histological subtype, grade, laterality, N stage, primary site surgery, lymph node surgery, other surgery, lung metastasis, and primary tumour size (Table 5). The C-index of the prediction result reached 0.617, which indicated weak consistence [15]. A nomogram was generated based on the multivariate-Cox analysis (Fig 2). The predicted value of the nomogram resembled the observed value, in which tumour size ≥ 1 cm, scirrhous and spindle cell variant subtype, and no primary site surgery demonstrated high risks, with a score >50, as shown in the nomogram. The risk scores of the patients were calculated and then divided into a high risk group and a low risk group according to the median risk score (1.032198). The low risk group had better survival outcomes than the high risk group (p<0.050, Fig 3A).

**Table 4:** Univariate-Cox regression analysis of the cohort survival.

Clinical Characteristics	HR	HR.95L	HR.95H	Pvalue
Age	1.026499	0.98239	1.072589	0.243167
Race	0.991507	0.944034	1.041367	0.733314
Sex	1.028881	0.957975	1.105035	0.434501
Year of_diagnosis	0.994404	0.978163	1.010914	0.504169
**Histologic_type**	**0.91162**	**0.845244**	**0.983207**	**0.016438**
**Grade**	**1.073388**	**1.051498**	**1.095734**	**1.62E-11**
Laterality	1.50432	0.782187	2.893142	0.221047
**T stage**	**1.046208**	**1.033332**	**1.059244**	**8.71E-13**
**N stage**	**1.077906**	**1.039928**	**1.11727**	**4.14E-05**
**Primary site surgery**	**2.782811**	**2.350981**	**3.293961**	**1.25E-32**
**Lymph node surgery**	**2.842242**	**2.027431**	**3.98452**	**1.36E-09**
**Other site surgery**	**1.540951**	**1.33266**	**1.781797**	**5.35E-09**
**Bone metastasis**	**1.084886**	**1.029385**	**1.143379**	**0.002359**
Brain metastasis	1.044228	0.941435	1.158244	0.413051
**Lung metastasis**	**0.768457**	**0.730299**	**0.808609**	**3.85E-24**
**Tumor_size**	**1.125901**	**1.092126**	**1.160721**	**2.33E-14**

**Table 5:** Multivariate-Cox regression analysis of the cohort survival.

Clinicopathological Characteristics	coef	Exp(HR)	lower0.95	upper0.95	Pr(>|z|)
**Age**					
Age<50	reference				
Age50-59	0.162	1.18	1.0414	1.3277	0.008925**
Age≥60	0.174	1.19	1.0593	1.3371	0.003385**
**Race**					
Black	reference				
White	-0.0312	0.969	0.8967	1.0478	0.432537
Other	-0.0156	0.985	0.8904	1.0887	0.761277
Unknown	-0.189	0.828	0.4871	1.4068	0.484953
**Sex**					
Female	reference				
Male	0.0311	1.03	0.9594	1.1093	0.400637
**Year of diagnosis**					
2010	reference				
2011	-0.0428	0.958	0.8668	1.059	0.402142
2012	0.00733	1.01	0.9134	1.111	0.883298
2013	-0.0325	0.968	0.8773	1.068	0.516716
2014	-0.0305	0.97	0.8782	1.0714	0.548106
2015	-0.0925	0.912	0.8248	1.0076	0.069973
**Histologic type**					
NOS	reference				
Fibrolamellar	-0.618	0.539	0.3527	0.824	0.004318**
Scirrhous	0.0522	1.05	0.4679	2.3724	0.899702
Spindle cell variant	0.566	1.76	1.1032	2.8135	0.017728*
Clear cell variant	-0.267	0.765	0.5393	1.0861	0.134265
**Grade**					
I	reference				
II	0.124	1.13	0.9813	1.3065	0.088888.
III	0.477	1.61	1.3963	1.8586	<0.001***
IV	0.565	1.76	1.3316	2.3259	<0.001***
Unknown	0.342	1.41	1.2478	1.5886	<0.001***
**Laterality**					
Paired	reference				
Not Paired	0.63	1.88	0.9732	3.6228	**0.040269.**
**T stage**					
T0	reference				
T1	-0.0985	0.906	0.6169	1.3311	0.615519
T2	-0.0411	0.96	0.6504	1.4162	0.835877
T3a	0.1	1.11	0.7526	1.6239	0.609127
T3b	0.144	1.16	0.7845	1.6996	0.465908
T3NOS	0.205	1.23	0.6061	2.4869	0.568891
T4	0.102	1.11	0.7496	1.6372	0.607582
TX	-0.0363	0.964	0.6525	1.4252	0.855385
**N stage**					
N0	reference				
N1	0.0993	1.1	1.0297	1.1844	0.005433**
NX	0.0183	1.02	0.9372	1.1067	0.666113
**Primary site surgery**					
Yes	reference				
No	0.809	2.25	1.8874	2.6737	<0.001***
**Lymph node surgery**					
Yes	reference				
No	0.588	1.8	1.2707	2.5505	<0.001***
**Other site surgery**					
Yes	reference				
No	0.206	1.23	1.0596	1.426	0.006445**
**Bone metastasis**					
Yes	reference				
No	0.0261	1.03	0.9615	1.0958	0.433311
Unknown	-0.0283	0.972	0.7913	1.1942	0.787558
**Brain metastasis**					
Yes	reference				
No	-0.153	0.859	0.7107	1.0373	0.113941
Unknown	-0.061	0.941	0.7169	1.2348	0.660246
**Lung metastasis**					
Yes	reference				
No	-0.33	0.719	0.6767	0.7643	<0.001***
Unknown	-0.367	0.693	0.5773	0.8319	<0.001***
**Tumour size**					
<1cm	reference				
≥1cm	1.03	2.79	1.0389	7.5086	0.041795*
Unknown	0.24	1.27	1.1715	1.3806	<0.001***

Significance codes: <0.001’***’ 0.001’**’ 0.01’*’ 0.05’.’

Concordance = 0.617 (se = 0.005)

Likelihood ratio test = 554.3 on 41 df, p = <2e-16

Wald test = 487.3 on 41 df, p = <2e-16

Score (logrank) test = 504.8 on 41 df, p = <2e-16

Regarding the different age groups, results showed that HR increased with age. Patients aged between 50 and 59 years (HR = 1.180, 95%CI:1.041–1.328, p<0.050) and age≥60 (HR = 1.190, 95%CI:1.060–1.337, p<0.050) had a worse prognosis than patients aged <50 years. Consistently, survival analysis suggested that patients aged 50–59 years and ≥ 60 years achieved similar survival outcomes, while those aged < 50 years had better probability of a longer OS (p<0.050, Fig 3B). Additionally, compared with other metastatic sites, the absence of lung metastasis was exclusively associated with a better prognosis (HR = 0.719, 95%CI: 0.677–0.764, p<0.001), which indicated that lung metastasis result in poorer prognosis; regarding different histological types, patients diagnosed with spindle cell type had the worst prognosis (HR = 1.760, 95%CI: 1.103–2.814, p<0.050) while fibrolamellar had the best prognosis (HR = 0.539, 95%CI: 0.353–0.824, p<0.050). Also, Kaplan-Meier curve confirmed these results (Fig 3C). However, among the 5274 samples, about 98% samples were NOS types, and other types were less. Effective statistical analysis cannot be performed due to the large difference in sample size. Therefore, the results should be further confirmed. Moreover, no primary-surgery-treated patients showed the second highest HR related to survival (HR = 2.250, 95%CI, 1.887–2.673, p<0.001), which may identify surgical treatment as the second most important factor affecting OS. Primary site surgery had a prominent effect on improving survival prognosis (p<0.050, Fig 3D). The most important factor affecting survival was tumour size≥1 (HR = 2.790, 95%CI, 1.039–7.509, p<0.050). However, due to the insufficiency of cases, there is only 3 tumours with size≥1cm. Therefore, this result is controversial. It is noteworthy that patients diagnosed in 2015 exhibited a lower risk of death compared to other years, which might be explained by the insufficiency of the follow-up period. However, this outcome was not statistically significant (p = 0.07).

Other statistically significant factors related to poorer prognosis included: no other site surgery (HR = 1.230, 95%CI, 1.057–1.426, p<0.010), no lymph node surgery (HR = 1.800, 95%CI, 1.271–2.551, p<0.001), N1 stage (HR = 1.100, 95%CI, 1.030–1.184, p<0.010), no paired laterality (HR = 1.880, 95%CI, 0.973–3.623, p<0.050), advanced grade (Grade III: HR = 1.610, 95%CI, 1.396–1.859, p<0.001; Grade IV: HR = 1.760, 95%CI, 1.332–2.326, p<0.001; unknown: HR = 1.760, 95%CI, 1.248–1.589, p<0.010). The reference of the HR can be found in Table 5.

### Kaplan-Meier analysis

**Fig 4:**
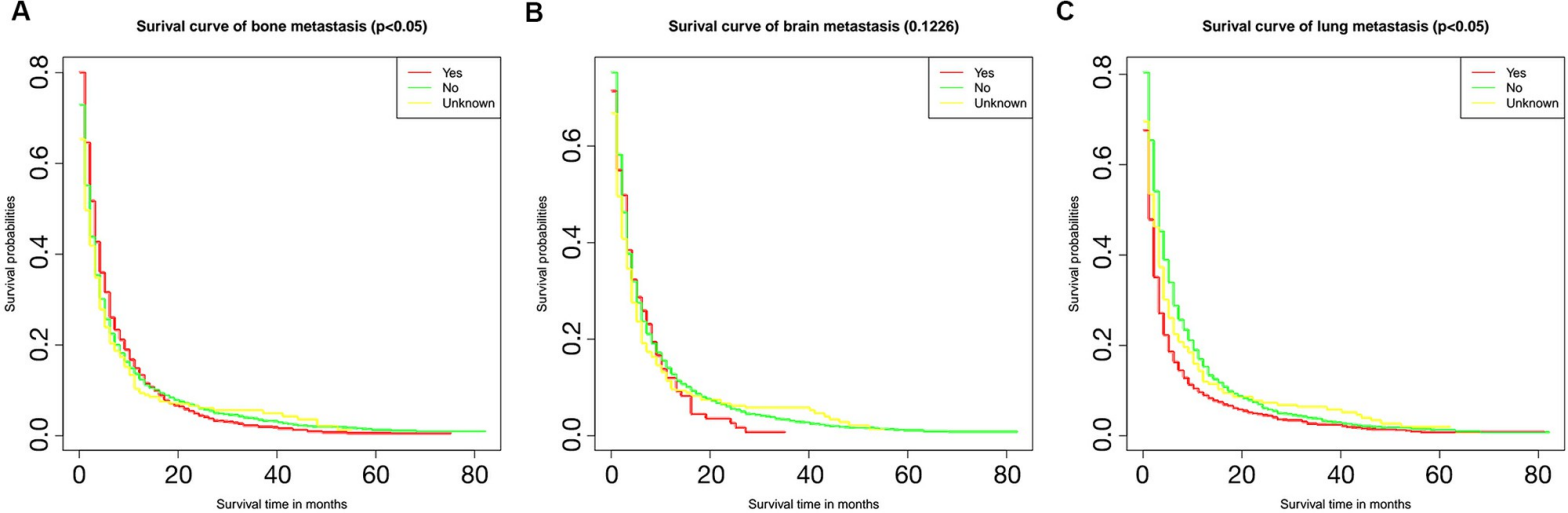
Kaplan-Meier curves among different metastasis status. The Kaplan-Meier survival curves show the differences on survival probabilities among metastasis, non-metastasis and unknown metastasis status at bone (A), brain (B) and lung (C).

**Fig 5:**
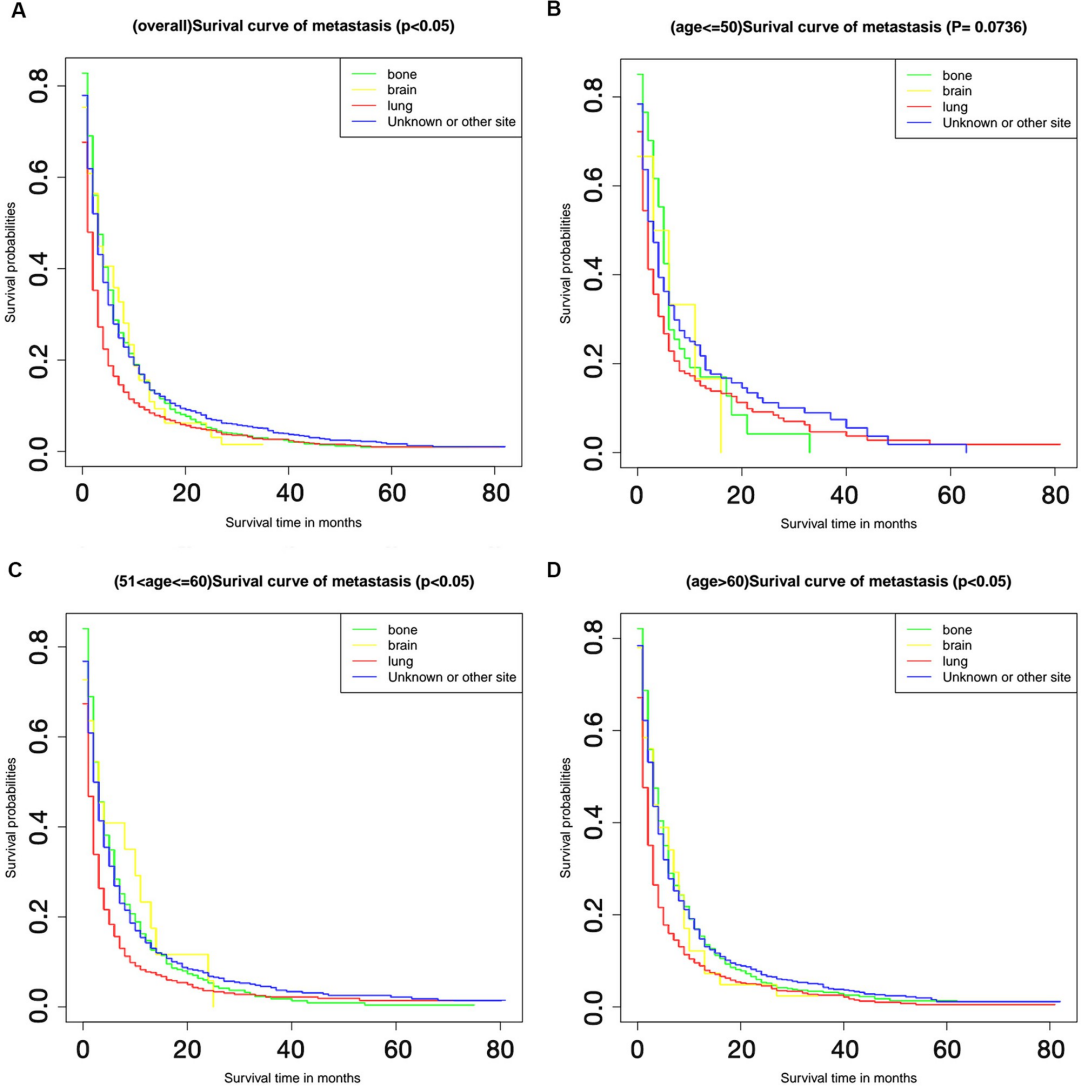
Kaplan-Meier curves for different age groups. The Kaplan-Meier survival curves show the differences on survival probabilities among bone metastasis, brain metastasis, lung metastasis and unknown metastasis site for all patients (A), patients < 50 years (B), patients in 50–59 years (C) and patients ≥ 60 years (D).

**Fig 6:**
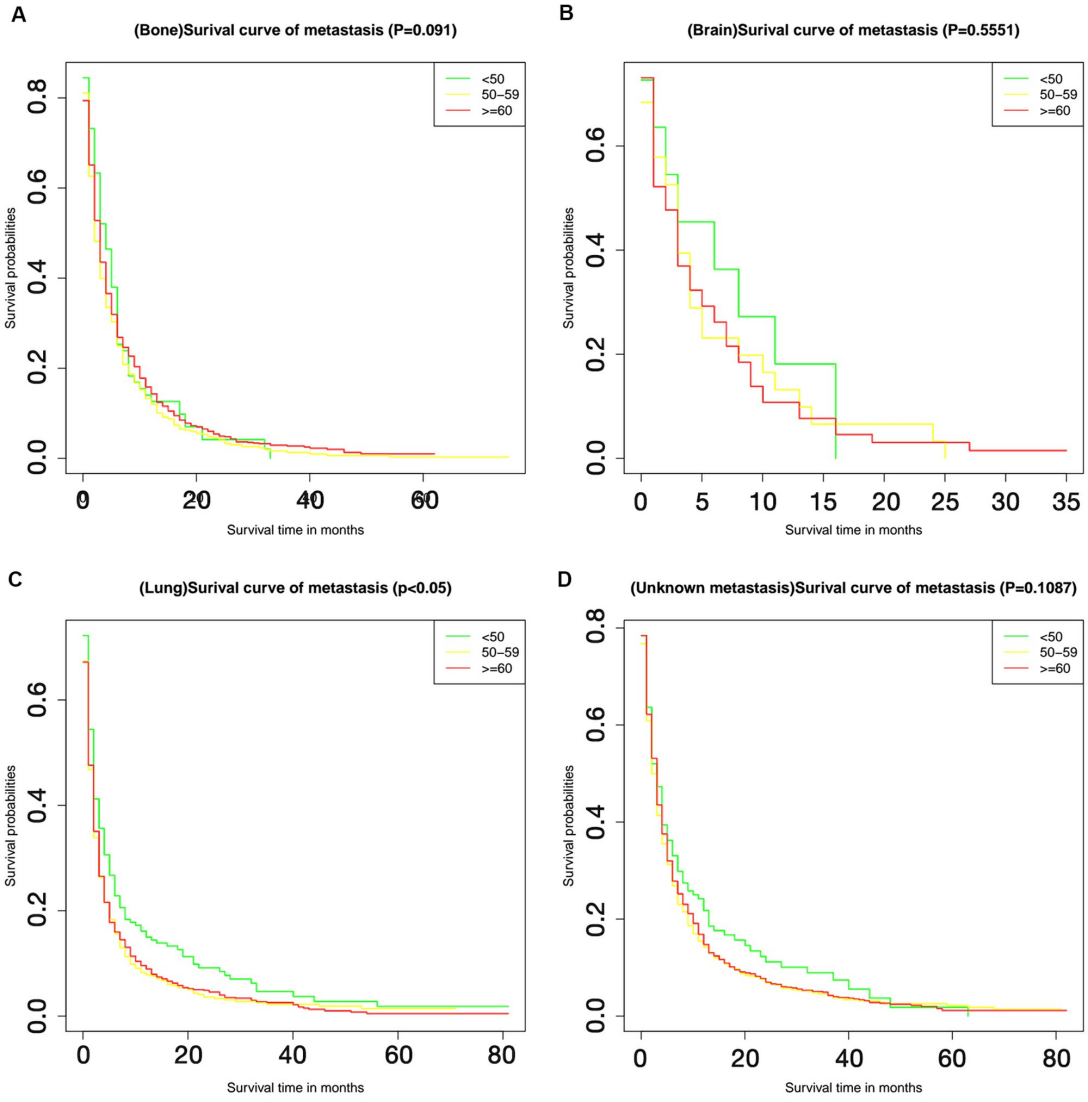
Kaplan-Meier curves for different metastatic sites. The Kaplan-Meier survival curves show the differences on survival probabilities among < 50 years, 50–59 years and ≥ 60 years patients with bone metastasis (A), brain metastasis (B), lung metastasis (C) and unknown metastasis (D).

The relationship between different metastatic sites and survival is shown in Fig 4. The unknown metastasis category included patients with intrahepatic metastasis or extrahepatic metastasis with an unknown location. The presence of metastasis at any of these sites was identified as a risk factor for death. Whereas bone metastasis exhibited better short-term prognosis than lung metastasis, within the 20 months follow-up period, but the long-term prognosis was worse. Additionally, the comparison of OS between patients from different age groups with different metastasis patterns is illustrated in Fig 5; while the comparison of OS between patients from different metastatic site groups with different ages, is shown in Fig 6. From Fig 5, lung metastasis exhibited worse prognosis within 20 months among all patients and in patients above the age of 60. In patients age 51–60, lung metastasis had the worst prognosis before approximate 40 months, overweighted by bone metastasis after 40 months. Shown by Fig 6, the prognosis of patients with lung metastasis showed significant correlation to the age. Patients younger than 50 had better prognosis than elderly patients (P < 0.05).

### What is the value of primary site surgery?

**Fig 7:**
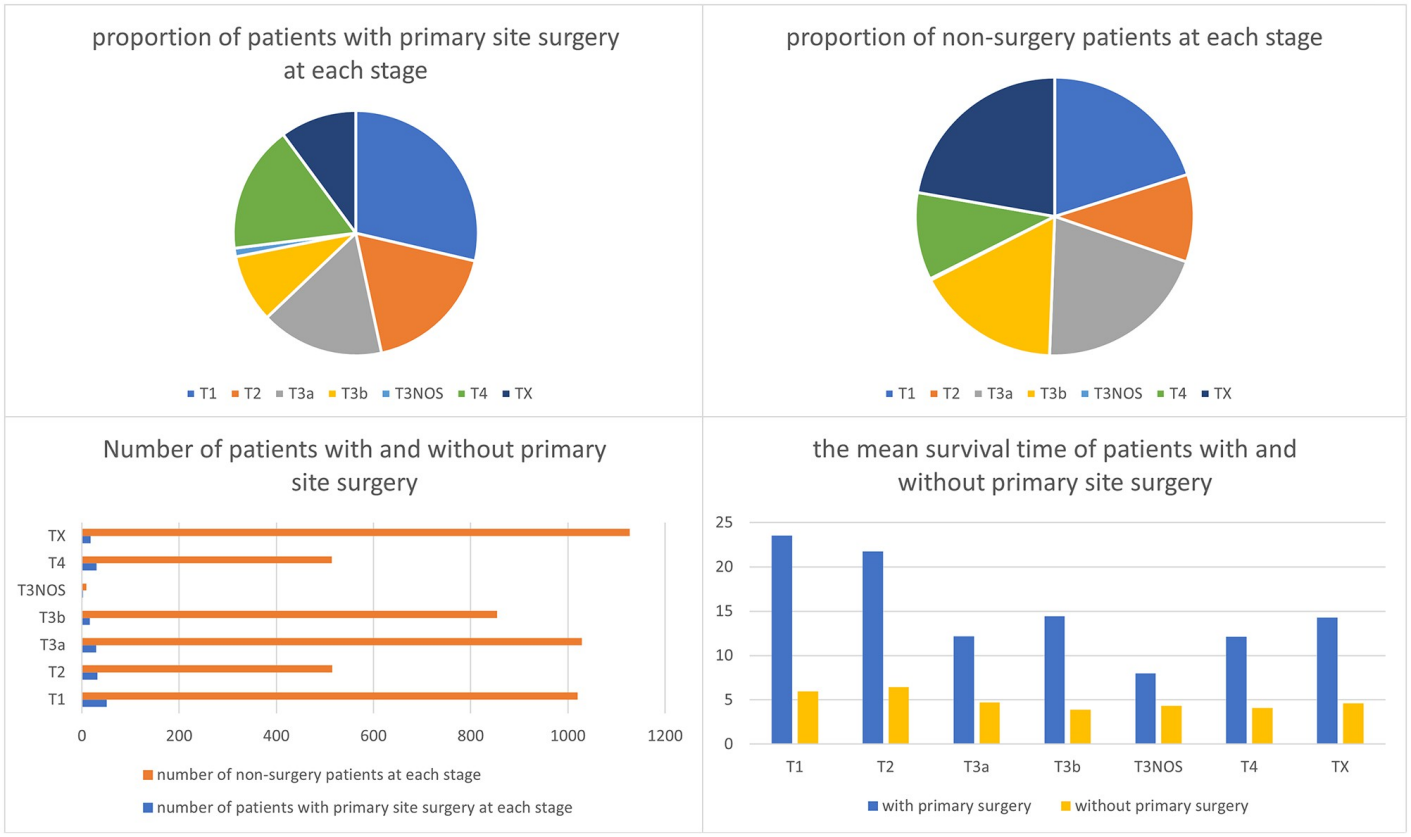
Outcome of the primary site surgery. In the bar chart of mean survival time (months), the bars representing patients undergone primary site surgery were on the left and non-primary site surgery were on the right.

As illustrated in Fig 7, except for the T0 stage patients, a primary site surgery was performed on patients at all stages, including advanced stages. In all stages of the disease, the overall mean survival of patients with a primary site surgery (mean = 17.4, sd = 17.6) seemed to surpass that of those without a primary site surgery (mean = 4.9, sd = 8.2). This is certified by our results of cox regression (Shown in Tables 4 and 5). Table 6 showed that there was a significant decrease in the number of patients undergoing surgery for patients with T3a and T3b tumors.

**Table 6:** Chi-square test of patients undergone primary site surgery at different T stages.

	PSS	NPSS	p value
T1	51	1020	
T2	32	515	0.4125
T3a	29	1029	0.0194
T3b	16	854	0.0007
T3NOS	2	9	0.1771
T4	30	514	0.593
TX	18	1127	<0.0001

Note: PSS, Patients with primary site surgery; NPSS, Patients with no primary site surgery.

## Discussion

In the present study, the correlation between metastatic patterns and age were investigated in metastatic HCC patients based on the data in SEER database. Age was identified as a risk factor for bone metastasis and death. Among the 5274 patients, 4281 were males (81.1%) and 993 were females (18.8%). It is a known fact that males are more often affected with liver cancer [16]. Multiple underlying mechanisms had been proposed to explain the gender disparity in live cancer. For example, Naugler et al. suggested that estrogen-mediated inhibition of interleukin-6 production by Kupffer cells (KCs) decreased the risk of liver cancer in women [16]. Manieri et al. indicated that the reduced levels of adiponectin was responsible for the elevated risk of liver cancer in male [17]. When analysing the different distant metastatic sites, lung metastases were associated with the poorest prognosis. We also verified that the lung was the leading site of distant metastasis. Previous studies reported that the most favourable sites for HCC extrahepatic metastasis were the lung, lymph nodes, bone, and adrenal gland, in that order [18, 19]. Although HCC is considered to have a lower metastatic rate than other solid tumours such as lung, breast, and colonic tumours, 25% of HCC patients will develop lung metastasis in 5 years [20]. According to our results, several factors were significantly correlated with an increase of both distant metastasis and mortality, including age ≥ 50 years, spindle cell variant, grade, no primary site surgery, no lymph node surgery, no other surgery, N1 stage, tumour size, and presence of lung metastases. In 2005, Natsuizaka M et al. reported that 73.8% patients with metastatic HCC, had HCC at T3 and T4 stages [21]. However, this was not observed in our present study. This difference may be explained by the fact that a more frequent use of advanced imaging methods may have helped detect liver cancer at an earlier stage.

Our results show that most cases of liver cancer with extrahepatic metastases were diagnosed in patients above the age of 50 years. Moreover, older patients were associated with a higher risk of distant metastases and an elevated mortality rate. This can be explained by immune infiltration and the formation of a unique microenvironment that is critical for tumorigenesis and metastasis in the liver [22, 23]. During the process of tumorigenesis, the accumulation of mutations in these hepatocytes, which is correlated to age, underpins the invasive behaviour of the tumour [13, 24–27]. Moreover, the liver is a highly metabolically active organ, and liver cancer is significantly related to the metabolic disruption occurring during chronic inflammation, with increased glycolysis and decreased TCA cycle activity [28–30]. Therefore, we believe that age-related mutations and age-related variations in metabolism may cause metastasis and death. Furthermore, this study illustrated that survival outcomes were correlated with the different metastatic sites independently. It has been demonstrated that cause of death in liver cancer patients is not usually attributed to extrahepatic metastatic lesions [19]. However, our study showed patients with lung metastases have a worse prognosis than other metastases patterns. This could be explained by the current insufficient methods for the management of lung metastasis. The lung metastasis group had the highest proportion of patients and was more likely to be diagnosed with other metastases. For this reason, the aforementioned outcomes suggest that physicians should be more aggressive when managing patients with lung metastasis.

The analysis revealed that patients with distant metastases who underwent surgery showed a significantly lower mortality rate. In particular, those who underwent primary site surgery showed reduced metastasis risk to bones and lungs, than those who did not. But this may also imply that the patients with bones or lungs metastases are more unlikely to choose surgical treatment. Patients at T1-2 stages have a 5-year survival rate of 50% [31]. According to the guidelines, surgery is the first-line option for early HCC, whereas it is only optimal for patients classified as very early stage, and as early stage only if certain conditions are present (e.g. well-preserved liver function, sufficient amount of parenchyma, optimal portal pressure, and acceptable for laparoscopic invasive approach) [8]. Lee et al. reported that the portal lymph node is one of the leading sites of primary liver cancer metastasis [12]. Nevertheless, there are controversies regarding whether surgery should be performed in patients at advanced stages, as a surgical procedure may stimulate regeneration, elevating the risk of recurrence and mortality. It has been reported that as many as 65% of patients experience recurrence within 5 years after surgery in patients at advanced stages [32]. These are potential hazards for relapse and postoperative death, in patients with decreased liver function. Our analysis verified the availability and importance of surgery in the treatment of liver cancer with distant metastasis in patients at all stages.

There were some limitations to this study. First, the limited data regarding co-morbidities and management provided by the SEER database, which might have introduced confounding factors affecting the prognosis of patients. Second, intrahepatic metastases and metastases to other sites were not included in the survival analysis. Third, patients with unknown metastatic patterns may have affected the results.Fourth, management and nursing should also be defined as indicators of prognosis for follow-up research. Lastly, among the 5274 samples, only 4 samples had tumor size ≥ 1cm, while 3730 samples had tumor size < 1cm. For histologic types, about 98% samples were NOS, and other types were less. Effective statistical analysis on tumor size and histologic types cannot be performed due to the large difference in sample size. Therefore, the statistical results on tumor size and histologic types were unreliable. Further effective statistical analysis analysis based on large sample size are needed to evaluate the results. the data provided by the database allowed the analysis of the metastatic patterns of primary liver cancer; however, further research is needed to obtain a better characterization of these patterns.

In conclusion, this population-based study revealed a potential correlation between metastatic patterns and clinical features such as age, histologic type, grade, and TNM stage. This study also defined that primary site surgery could significantly reduce the risk of metastasis and extend overall survival. This indicates that patients diagnosed with N1 stage, spindle cell variant, or higher-grade cancer may benefit more from surgery than what is currently believed. Moreover, a considerable number of elderly patients were not prescribed satisfactory surgery (Among the 5274 patients, the majority of patients did not receive surgery. For primary site surgery: 97%; for lymph node surgery: 99%; and for other surgery: 96%), of which those aged ≥ 60 years had the highest mortality rate. Many liver cancer patients are diagnosed at an advanced stage with distant metastasis; thus, these findings will improve physicians’ ability to manage liver cancer patients with a more personalised approach.
